# Independent and joint correlation of *PNPLA3* I148M and *TM6SF2* E167K variants with the risk of coronary heart disease in patients with non-alcoholic fatty liver disease

**DOI:** 10.1186/s12944-020-01207-9

**Published:** 2020-02-24

**Authors:** Jian-Ting Wu, Shou-Sheng Liu, Xiang-Jun Xie, Qun Liu, Yong-Ning Xin, Shi-Ying Xuan

**Affiliations:** 1 Department of Gastroenterology, Qingdao Municipal Hospital, Qingdao University, Qingdao, 266011 China; 2 Central Laboratories, Qingdao Municipal Hospital, Qingdao University, Qingdao, 266071 China; 3 Digestive Disease Key Laboratory of Qingdao, Qingdao, 266071 China; 4 Department of Infectious Disease, Qingdao Municipal Hospital, Qingdao University, Qingdao, 266011 China

**Keywords:** *PNPLA3* I148M, *TM6SF2* E167K, Non-alcoholic fatty liver disease, Coronary heart disease

## Abstract

**Background:**

CHD is reported to be the primary cause of death in patients with NAFLD. Genetic susceptibility genes contribute to the developmental risk of NAFLD or CHD. Whether the genetic factors could affect the risk of CHD in NAFLD patients is not clear. The aim of this study was to investigate the association of *PNPLA3* I148M and *TM6SF2* E167K variants with the risk of CHD in NAFLD patients in Chinese Han population.

**Patients and methods:**

*PNPLA3* I148M and *TM6SF2* E167K variants were genotyped in a cohort of 189 patients with NAFLD and CHD, as well as 242 patients with NAFLD and 242 healthy controls by gene sequencing. Additionally, serum lipids profiles were determined by standard clinical laboratory methods.

**Results:**

The minor allele frequency of *PNPLA3* I148M and *TM6SF2* E167K were 0.39 and 0.06 in this cohort, respectively. The distributions of *PNPLA3* I148M genotypes and alleles were significant different in NAFLD group vs controls and in NAFLD+CHD group vs NAFLD group (all *P* <  0.05). NAFLD patients who carry the CG + GG genotype suffered the relative lower risk of CHD than CC genotype carriers (OR = 0.6, 95%CI: 0.40–0.90, *P* = 0.01). In addition, *PNPLA3* I148M and *TM6SF2* E167K possess the joint correlation with the decreased risk of CHD in NAFLD patients with the increased number of risk alleles. Besides, *PNPLA3* I148M and *TM6SF2* E167K variants associated with the decreased serum lipid levels in overall series.

**Conclusions:**

There was a joint protective correlation of *PNPLA3* I148M and *TM6SF2* E167K variants with the developmental risk of CHD in NAFLD patients. *PNPLA3* I148M and *TM6SF2* E167K variants might correlated with the decreased risk of CHD in NAFLD patients by associated with the reduced serum lipid levels.

## Background

Non-alcoholic fatty liver disease (NAFLD) is becoming the leading cause of chronic liver disease in the world [1]. In China, the prevalence of NAFLD was about 25% and has approximately doubled in the past two decades [2, 3]. The typical characteristics of NAFLD patients include obesity, insulin resistance, higher serum lipid profiles, and abundant of NAFLD patients possess the diabetes and hyperlipemia [4]. In Epidemiological studies, NAFLD had been proven as an increased risk of atherosclerosis and cardiovascular disease. Some experts even proposed that NAFLD is the causal risk of coronary heart disease (CHD), despite both the risk factors of them were different [5, 6]. Therefore, exploring the CHD risk in NAFLD patients and identifying the risk factors are necessary to manage and prevent the development of CHD in NAFLD patients.

Genetic factor is the significant risk factors for the development of NAFLD, which had been studies for many years [7]. In 2008, Romeo et al. firstly reported the key role of *PNPLA3* I148M in NAFLD patients by genome-wide association study (GWAS), which described that *PNPLA3* I148M variant conferred to the higher hepatic fat content and severe hepatic inflammation [8]. In 2014, Kozlitina et al. founded that *TM6SF2* E167K was another significant polymorphism site for the risk of NAFLD by GWAS [9]. Subsequently, many studies had investigated the roles of *PNPLA3* I148M and *TM6SF2* E167K in different countries and ethnic, and the results also proven that *PNPLA3* I148M and *TM6SF2* E167K variants were the risk factors for the development of NAFLD [10–13]. In addition, *PNPLA3* I148M and *TM6SF2* E167K variants were found to have additive effect on increasing the NAFLD risk in Chinese Han population [14, 15]. Recently, some studies revealed the independent effect of *PNPLA3* I148M and *TM6SF2* E167K on the risk of cardiovascular disease. The positive association of PNPLA3 I148M with the premature CHD in T2DM patients was observed, and Ruschenbaum et al. found that *PNPLA3* I148M is associated with a relatively benign CHD risk in German. Pirola conducted a meta-analysis to investigate the role of *TM6SF2* E167K in patients with NAFLD and CHD, and they found that *TM6SF2* E167K may possess the dual and opposite role in protecting against CHD and increase the risk of NAFLD [16–18].

In consideration of the tightly association of NAFLD and CHD in patients, and *PNPLA3* I148M and *TM6SF2* E167K variants may possess the joint effect to conferred to the higher risk of NAFLD, it is meaningful to investigate the correlation of *PNPLA3* I148M and *TM6SF2* E167K variants with the development risk of CHD in NAFLD patients. The aim of this study was to explore the relationship of *PNPLA3* I148M and *TM6SF2* E167K variants with the risk of CHD in NAFLD patients in Chinese Han population, and explore the effects of *PNPLA3* I148M and *TM6SF2* E167K variants on the serum lipid profiles in overall series.

## Patients and methods

### Study subjects

This study was conducted according to the principles of Helsinki declaration and its appendices [19], and were approved by the Ethical Committee of Qingdao Municipal Hospital (Qingdao, China). All the subjects had signed the informed consent before participating in this study. All the subjects were recruited from June 2018 to March 2019, which include 266 healthy controls, 242 NAFLD patients diagnosed by B-type ultrasonography, and 189 patients with NAFLD and CHD (NAFLD+CHD) who undergone elective coronary angiography. All the subjects were Chinese Han population who had the normal diet and moderate exercise. The age and sex of all the recruited subjects were matched. Subjects as below were excluded: 1) possessing a history of heart failure; 2) long-term consumption of alcohol (males > 210 g/w, females > 140 g/w) or cigarettes; 3) accompanied with the viral hepatitis, autoimmune hepatitis, drug-induced hepatitis, or various liver cirrhosis; 4) suffering from tumors or surgery in nearly 2 years; 5) undergone any treatment for NAFLD or CHD.

### Biochemical analyses

Blood samples of each subject were taken after a 12-h overnight fasting and placed into an ethylene diamine tetraacetic acid (EDTA)-containing tube. The serum clinical parameters such as triglyceride (TG), total cholesterol (TC), low-density lipoprotein (LDL), high-density lipoprotein (HDL), Alanine aminotransferase (ALT), aspartate aminotransferase (AST), gamma-glutamyltranspeptidase (GGT), alkaline phosphatase (ALP), fasting plasma glucose (FPG), total bilirubin (TBIL) were measured by standard clinical laboratory techniques. The basic information such as name, gender, age, body height and weight were acquired by a standard study questionnaire. Body mass index (BMI) was calculated as the mass (kg)/height (m)^2^.

### Genomic DNA extraction and genotyping

Genomic DNA was extracted from blood samples with the DNA extraction Kit (TIANGEN, Beijing, China) and stored at -20 °C until use. Polymerase chain reaction (PCR) was used for the genotyping of PNPLA3 I148M and TM6SF2 E167K with the primers: 5′-AACTTCTCTCTCCTTTGCTTTCACA-3′ and 5′- GGAGGGATAAGGCCACTGTAGA-3′ for PNPLA3 I148M; 5′- TGTCTCAGAACAAACAAACAAACAGA-3′ and 5′- GTAGGGGATGGTGAGGAAGAAG − 3′ for TM6SF2 E167K. The PCR amplification reaction was conducted as the following procedure: 95 °C for 5 min, 40 cycles before denaturation at 94 °C for 30 s, annealing at 58 °C for 30 s and elongation 30 s at 72 °C. The genotypes of *PNPLA3* I148M and *TM6SF2* E167K variants were detected by ABI3730XL (Foster City, CA, USA) and then calculated by Gene Mapper 4.1 software.

### Statistical analysis

SPSS 24.0 statistical software was used for statistical analysis. The Hardy-Weinberg equilibrium was measured using χ^2^ test. The difference of Genotype and allele distributions in each group was assessed by χ^2^ test and Fisher’s exact test where appropriate. Associations between the *PNPLA3* I148M, *TM6SF2* E167K and NAFLD+CHD patients were assessed in logistic regression models. SNPs were analyzed using an additive model (by coding the genotype 0, 1 and 2 for CC, CG and GG respectively) for *PNPLA3* gene and a dominant model (by coding the genotype 0 and 1 for CC and CT + TT respectively) for TM6SF2 gene. The lipids characteristics among each group were shown as mean ± SD and the differences was examined using student’s t test. The *P* values less than 0.05 were considered to be statistically significant.

## Results

### Clinical characteristics of the study subjects

Table 1 shows the characteristics of healthy controls, NAFLD patients and NAFLD+CHD patients. As the results shown, the age and sex of subjects in each group were all matched. The BMI values and serum AST levels in NAFLD+CHD patients were higher than in NAFLD patients and health controls, and the BMI values and serum AST levels in NAFLD group was also higher than in health controls (all *P* <  0.05). There were significant difference of serum FPG and HDL levels in NAFLD+CHD patients compared to NAFLD patients or health controls (all *P* < 0.05), but no obviously difference was observed between NAFLD patients and health controls (*P* > 0.05). In addition, serum ALT and TG levels in NAFLD+CHD patients and NAFLD patients were significantly higher than health controls (all *P* < 0.05).

**Table 1:** Clinical and biochemical characteristics of each group ^a^

Characteristic	NAFLD+CHD (*n* = 189)	NAFLD (*n* = 242)	Control (*n* = 266)
Age, y	58.10 ± 6.95	57.7 ± 8.53	58.90 ± 5.53
BMI, kg/m^2^	25.69 ± 3.2^*@^	26.76 ± 2.82^*^	23.65 ± 3.50
FPG, mmol/L	5.81 ± 1.81^*@^	5.00 ± 1.35	4.85 ± 1.55
ALT, U/L	30.23 ± 2.80^*^	34.56 ± 26.27^*^	22.47 ± 21.14
AST, U/L	45.73 ± 70.71^*@^	28.45 ± 21.49^*^	22.52 ± 11.84
TC, mmol/L	5.22 ± 1.17	5.48 ± 0.83^*^	5.10 ± 1.17
TG, mmol/L	2.00 ± 1.07^*^	1.92 ± 1.31^*^	1.52 ± 1.10
LDL, mmol/L	3.09 ± 1.06	3.26 ± 0.59	3.15 ± 0.73
HDL, mmol/L	1.03 ± 0.28^*@^	1.23 ± 0.21	1.29 ± 0.38

Abbreviations: *NAFLD* non-alcoholic fatty liver disease, *CHD* coronary heart disease, *BMI* body mass index, *FPG* fasting plasma glucose, *TC* total cholesterol, *TG* triglyceride, *LDL* low-density lipoprotein cholesterol, *HDL* high-density lipoprotein cholesterol, *AST* aspartate aminotransferase, *ALT* alanine aminotransferase

* Compared with the control group, *P* < 0.05; ^@^ compared with NAFLD group, *P* < 0.05

^a^Data are presented as mean ± SD

### Genotype and allele distributions of *PNPLA3* I148M and *TM6SF2* E167K

The genotype distributions of the two SNPs (*PNPLA3* I148M and *TM6SF2* E167K) were conforming to the Hardy-Weinberg equilibrium in each group (*P*_NAFLD + CHD_ = 0.92, 0.98; *P*_NAFLD_ = 0.99, 0.63; *P*_control_ = 0.87, 0.60, respectively). The minor allele frequency (MAF) of *PNPLA3* I148M and *TM6SF2* E167K genotyped in our study were comparable to the overall frequency in east Asians, all the allele frequency information were collected from 1000 Genomes Project [20] (Table 2).

**Table 2:** Minor allele frequency in the present study and other populations

SNP	Present study	AFR	AMR	EAS	EUR
*PNPLA3* I148M	0.39	0.12	0.48	0.35	0.23
*TM6SF2* E167K	0.06	0.02	0.06	0.09	0.07

Abbreviation: *AFR* African, *AMR* American, *EAS* East Asian, *EUR* European

### Independent correlation of PNPLA3 I148M with the risk of CHD in NAFLD patients

Table 3 demonstrated the genotype and allele distributions of *PNPLA3* I148M in NAFLD+CHD patients, NAFLD patients and healthy controls. As the results shown, the genotype and allele distributions of PNPLA3 I148M were significant different in NAFLD+CHD vs NAFLD groups and in NAFLD vs control groups. In the Table 4, the results showed that the genotype CG + GG could increase the risk of NAFLD significantly compared to controls (OR = 1.53, 95%CI: 1.06–2.22, *P* = 0.02). However, the CG + GG genotype could decrease the risk of CHD in NAFLD patients (OR = 0.60, 95%CI: 0.40–0.90, *P* = 0.01), which suggested the protective role of PNPLA3 I148M for the risk of CHD in NAFLD patients.

**Table 3:** Genotype and allele frequency of *PNPLA3* I148M in each group

SNP	NAFLD+CHD (n = 189)	NAFLD (*n* = 242)	Control (*n* = 266)	NAFLD+CHD vs NAFLD		NAFLD+CHD vs Control		NAFLD vs Control	
				χ^2^	*P* value	χ^2^	*P* value	χ^2^	*P* value
PNPLA3 I148M									
Genotype				9.30	0.01	0.21	0.90	8.36	0.02
CC	79	73	106						
CG	89	121	128						
GG	21	48	32						
Allele				9.13	0.00	0.09	0.77	4.69	0.03
C	247 (65.34)	267 (55.17)	340 (63.90)						
G	131 (34.66)	217 (44.83)	192 (36.09)

**Table 4:** Association between *PNPLA3* I148M and CHD outcome in NAFLD patients

*PNPLA3* I148M	NAFLD+CHD vs NAFLD		NAFLD+CHD vs control		NAFLD vs control	
	OR (95%CI)	*P* value	OR (95%CI)	*P* value	OR (95%CI)	*P* value
CC	1		1		1	
CG + GG	0.60 (0.40–0.90)	0.01	0.92 (0.63–1.35)	0.68	1.53 (1.06–2.22)	0.02

### Additive correlation of PNPLA3 I148M and TM6SF2 E167K with the risk of CHD in NAFLD patients

**Fig. 1:**
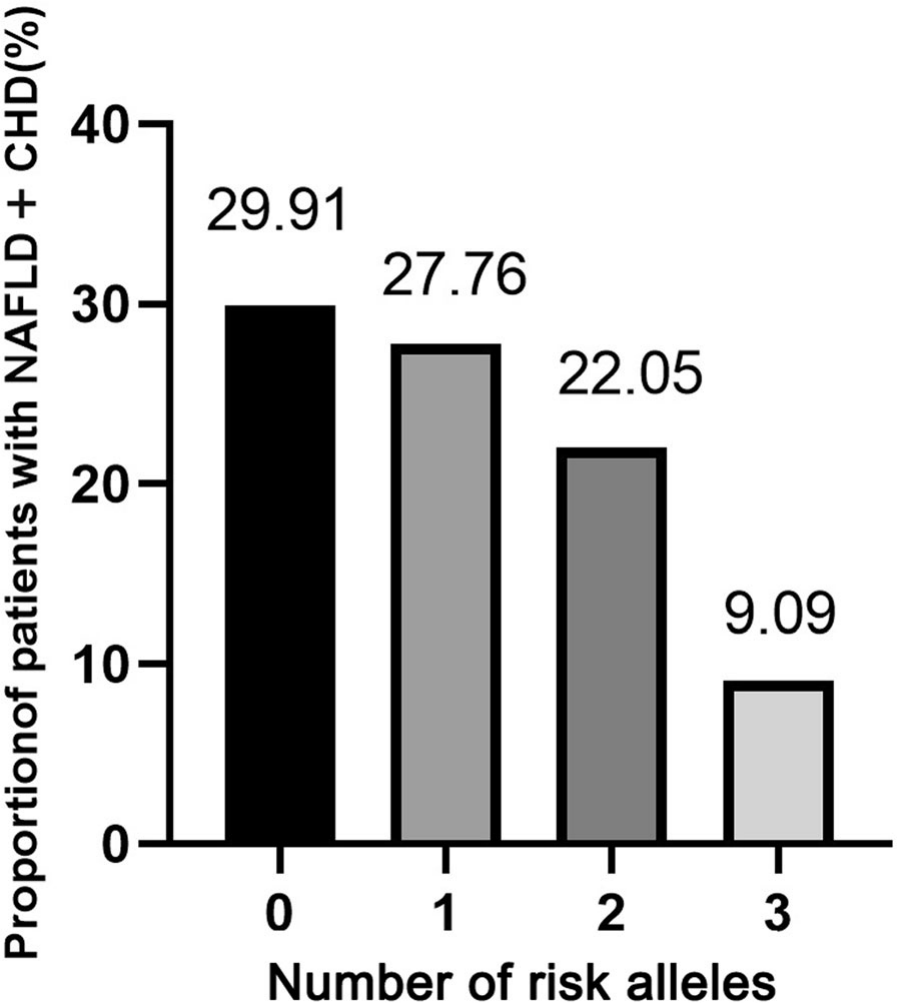
Proportion of patients with NAFLD+CHD in each group of subjects that harboring different number of risk alleles

We further investigated the join correlation of PNPLA3 I148M and TM6SF2 E167K with the outcome of CHD in NAFLD patients. Figure 1 showed the proportion of the patients with NAFLD and CHD harboring different number of risk alleles. In our study, we didn’t observe any individual with four SNPs. The proportion of patients with NFALD+CHD decreased significantly along with the increase of the number of risk alleles (χ^2^ = 270, *P* < 0.01). As the Table 5 shown, the increase of the number of risk alleles showed a strong protective correlation with the CHD risk in NAFLD patients.

**Table 5:** Risk for CHD outcome in NAFLD patients with different number of SNPs

Number of risk alleles	OR (95%CI)	*P* value
0	1	
1	0.632 (0.405–0.987)	0.04
2	0.418 (0.235–0.743)	< 0.01
3	0.119 (0.014–1.000)	0.05

### Metabolic traits of PNPLA3 I148M and TM6SF2 E167K

**Fig. 2:**
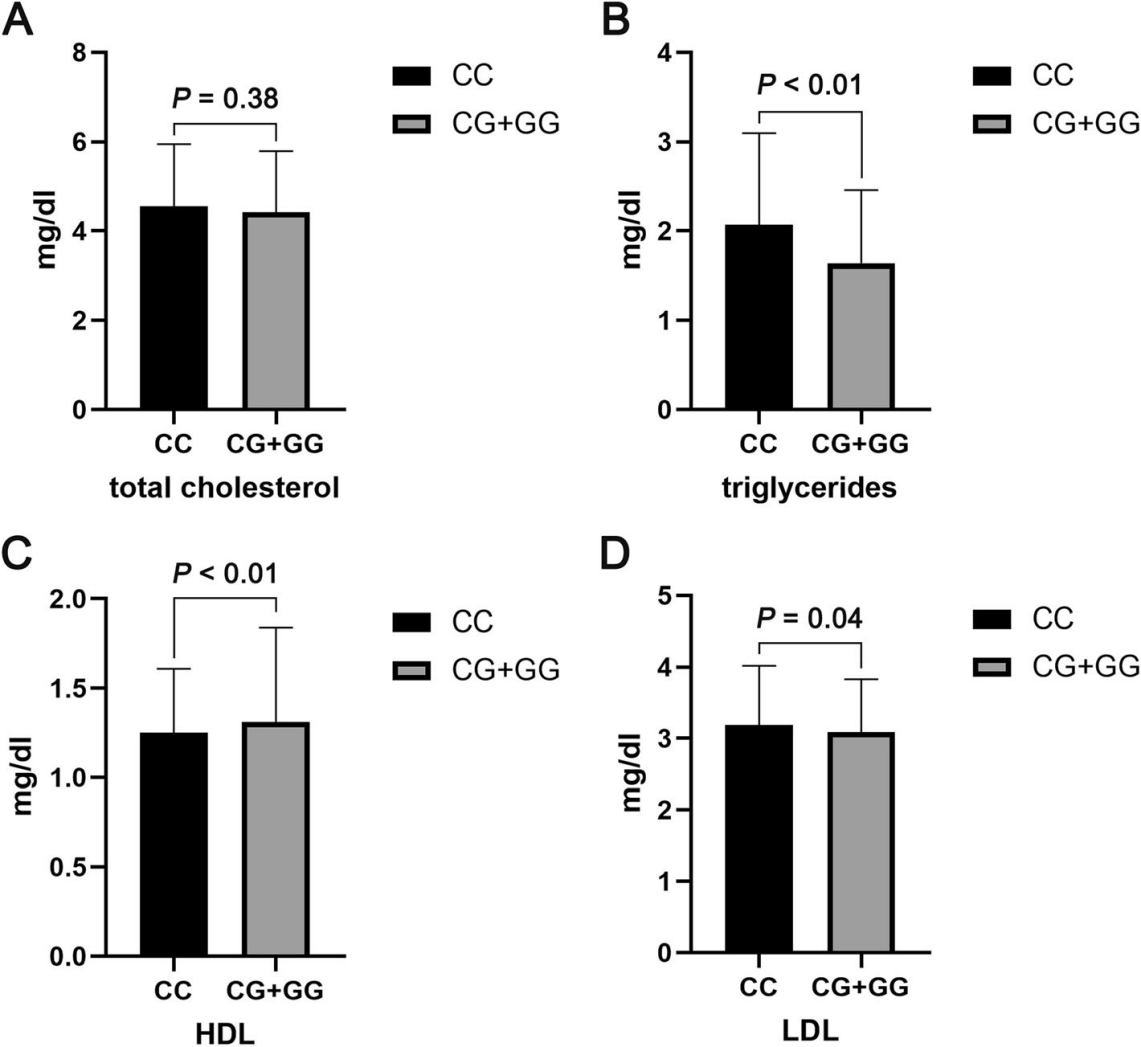
Differences of serum levels of (**a**) TC, (**b**) TG, (**c**) HDL, and (**d**) LDL between subjects with PNPLA3 CC genotype and CG + GG genotype. Data are presented as mean values and standard deviations (Mean ± SD)

**Fig. 3:**
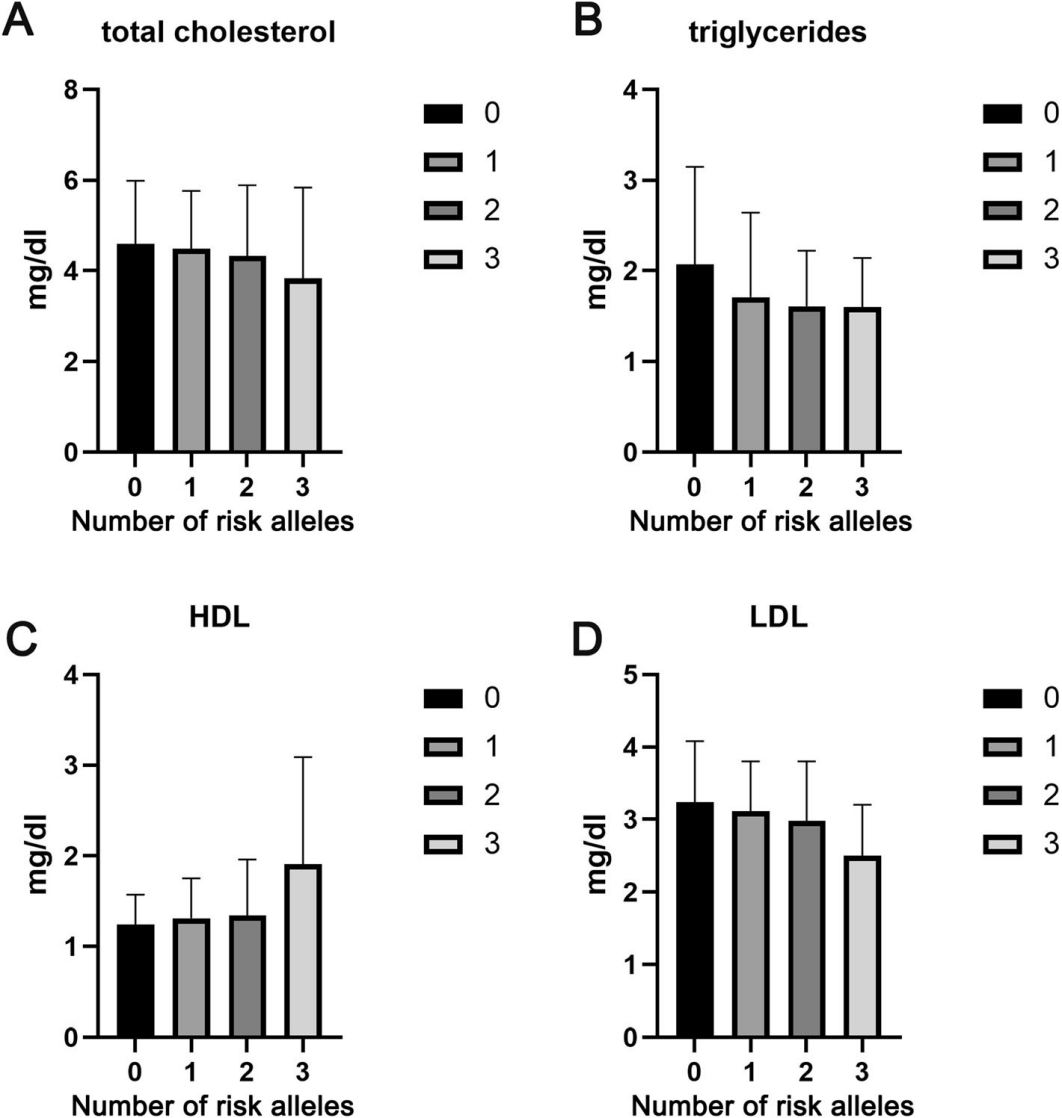
Differences of serum levels of (**a**) TC, (**b**) TG, (**c**) HDL, and (**d**) LDL between subjects with different number of risk alleles. Data are presented as mean values and standard deviations (Mean ± SD)

The difference of serum lipid levels between subjects with PNPLA3 CC genotype and CG + GG genotype was shown in the Fig. 2. We found that subjects with CC genotype possess the higher levels of TG and LDL and the lower level of HDL (all *P* < 0.05). No significant difference of TC level was observed between the two groups (*P* > 0.05). We further analyzed the serum levels of TC, TG, HDL, and LDL in subjects with different number of risk alleles. As shown in the Fig. 3, the serum levels of TG and LDL were decreased significantly with the increase of number of risk alleles. In addition, the serum level of HDL was increased significantly with the increase of number of risk alleles. No obvious difference was observed of the serum TC level in each group of subjects that harboring different number of risk alleles.

## Discussion

Previous epidemiological studies reported that NAFLD was associated with an increased risk of CHD, thereby put forward the hypothesis that NAFLD might be a causal risk factor for CHD [5, 6], In addition, CHD is the primary cause of death in patients with NAFLD [21]. To identify the CHD risk in NAFLD patients might be helpful in improving the management of NAFLD and CHD. Previous study had proven that PNPLA3 I148M and TM6SF2 E167K variants possess the additive effect on the risk of NAFLD [15]. In this study, we investigated the association of PNPLA3 I148M and TM6SF2 E167K variants with the risk of CHD in NAFLD patients for the first time. We counted the Minor allele frequency of PNPLA3 I148M and TM6SF2 E167K variant in this study, and the results were accorded with the mutation rate in East Asian. According to the results, PNPLA3 I148M variant was significant associated with the decreased risk of CHD in NAFLD patients. The possible reason is that PNPLA3 I148M could decrease the serum lipids levels in NAFLD, thereby lead to the decreased risk of CHD.

Several studies had shown that PNPLA3 rs738409 G allele might be associated with a relatively benign cardiovascular risk due to the tightly association with lower serum LDL level, which is the high risk factor for cardiovascular [9, 22]. In this study, we analyzed the correlation of PNPLA3 I148M variant with the serum lipid levels in overall series. We found that the serum levels of TG and LDL were significantly lower and the serum HDL levels were higher in subjects with CC genotype compared to CG + GG genotype carriers. These results suggested that PNPLA3 CG + GG genotype might tightly associate with the decreased serum lipid levels in overall series, which in accordance with the previous report [22]. In addition, some reports proposed that TM6SF2 E167K could decrease the plasma levels of LDL and TC, therefore, we also investigate the joint correlation of PNPLA3 I148M and TM6SF2 E167K variant with the risk of CHD in NAFLD patients and serum lipid levels in overall series. We found the risk of CHD in NAFLD patients was decreased along with the increase of risk alleles. In the overall series, the proportion of patients with NAFLD+CHD was decreased along with the increase of risk alleles of PNPLA3 I148M and TM6SF2 E167K. The serum TG and LDL levels were reduced, and serum HDL levels were decreased along with the increase of risk alleles. These results suggested that PNPLA3 I148M and TM6SF2 E167K possess the joint correlation with the decreased risk of CHD in NAFLD patients, and were associated with the decreased serum lipid levels in overall series.

There were some limitations in this study. Firstly, ultrosonography was performed to diagnosis of NAFLD other than liver biopsy. Secondly, the size of subjects in this study may not sufficiently large to explore the association between PNPLA3 I148M and TM6SF2 E167K with the risk of CHD in NAFLD patients. Thirdly, previous studies had showed that PNPLA3 I148M and TM6SF2 E167K variants were strongly associated with the decreasing kidney function, which impacts on the development of CHD [23–25], this factor was not taken into consideration.

## Conclusion

In summary, we investigated the correlation of PNPLA3 I148M and TM6SF2 E167K variants with the risk of CHD in NAFLD patients. The PNPLA3 I148M was associated with the decreased risk of CHD in NAFLD patients, and was associated with the decreased the serum lipid levels in overall series. PNPLA3 I148M and TM6SF2 E167K variants were tightly correlated with the decreased risk of CHD in NAFLD patients and the decreased serum lipid levels in overall series along with the increased numbers of risk alleles. These results suggested that genetic factor such as PNPLA3 I148M and TM6SF2 E167K are very important in the developmental risk of CHD in NAFLD patients, which could be regarded as the potential diagnostic biomarkers for the risk assessment of CHD in NAFLD patients.

## Data Availability

The data and materials are available from the corresponding author for the reasonable request.

## Abbreviations

ALP: Alkaline phosphatase
ALT: Alanine aminotransferase
AST: Aspartate aminotransferase
BMI: Body mass index
CHD: Coronary heart disease
CI: Confidence intervals
EDTA: Ethylene diamine tetraacetic acid
FPG: Fasting plasma glucose
GGT: Gamma-glutamyltranspeptidase
GWAS: Genome-wide association studies
HDL: High density lipoprotein
LDL: Low density lipoprotein
NAFLD: Non-alcoholic fatty liver disease
ORs: Odds ratios
PCR: Polymerase chain reaction
SNP: Single nucleotide polymorphism
TBIL: Total bilirubin
TC: Total cholesterol
TG: Triglycerides
TRIB1: Tribbles-1
